# Supplementary material for: Content-rich biological network constructed by mining PubMed abstracts
Source: BMC Bioinformatics. 2004 Oct 8;5:147. doi: 10.1186/1471-2105-5-147 (PMC528731; doi:10.1186/1471-2105-5-147)
Supplement: Additional File 2 — The original results of the above study (non-essential files are deleted to keep the file size under the limit set by BMC bioinformatics). [file 1471-2105-5-147-S2.bz2 › chilibotAdditionalFile2/dip10/656ID10449718E11047/html/TRPE_TRPG.html]

 


 **TRPE** and **TRPG** 
  
Found 35 abstracts in PubMed, retrieved 10.  
 

 What does Google say? 
 PDF only 
| .edu only 

---

**Interactive relationship** (e.g. stimulation, inhibition, etc)

**Neutral relationship**- It is a heterotetramer of anthranilate synthase  **TrpE**  and glutamine amidotransferase  **TrpG**  subunits, in which two  **TrpG**   **TrpE**  protomers associate mainly via the  **TrpG**  subunits.  Ref: 10449718 Proc Natl Acad Sci U S A, 1999
- The structure suggests a model in which chorismate binding triggers a relative movement of the two domain tips of the  **TrpE**  subunit, activating the  **TrpG**  subunit and creating a channel for passage of ammonia toward the active site of the  **TrpE**  subunit.  Ref: 10449718 Proc Natl Acad Sci U S A, 1999

**Non-interactive relationship** (e.g. studied together, co-existance, homology, etc.)

- Tk  **trpE**  and Tk  **trpG** , the genes that encode the two subunits of anthranilate synthase from the hyperthermophilic archaeon Thermococcus kodakaraensis KOD1, have been expressed independently in Escherichia coli.  Ref: 11237738 Biochem Biophys Res Commun, 2001
- In bacteria, the first reaction of the tryptophan biosynthetic pathway involves the conversion of chorismate and glutamine to anthranilate by the action of anthranilate synthase, which is composed of the alpha  **trpE**  gene product and beta  **trpG**  gene product subunits.  Ref: 12634337 Microbiology, 2003
- The anthranilate synthase complex Tk AS complex was obtained by heat treatment of the mixture of cell free extracts containing each recombinant protein, Tk  **TrpE**  alpha subunit and Tk  **TrpG**  beta subunit, at 85 degrees C for 10 min.  Ref: 11237738 Biochem Biophys Res Commun, 2001
- Nucleotide sequences upstream of  **trpE**  and  **trpG**  encode putative leader peptides similar in sequence to leader peptides found in other bacterial species, and the phenotypes of the mutants strongly suggest that transcription of these operons is regulated solely by attenuation.  Ref: 11325956 J Bacteriol, 2001
- A systematic polymerase chain reaction PCR analysis of P. aeruginosa strains that produce various subtypes of R and F pyocins revealed that the genes for every subtype are located between  **trpE**  and  **trpG**  in the same or very similar gene organization as for R2 and F2 pyocins, but with alterations in genes that determine the receptor specificity.  Ref: 11069649 Mol Microbiol, 2000
- The nucleotide sequence of a 2 4 kb BamHI SalI fragment of Streptomyces venezuelae ISP5230 DNA that complements  **trpE**  and  **trpG**  mutations in Escherichia coli contains two ORFs.  Ref: 9695930 Microbiology, 1998
- The plasmid population was heterogeneous with respect to the number of tandem repeats of a 1.8 kb unit carrying repAC1,  **trpG** , and remnants of  **trpE** .  Ref: 9872768 Appl Environ Microbiol, 1999
- The estA gene showed high similarity to an open reading frame of unknown function located in the  **trpE**   **trpG**  region of P. putida and to a gene encoding an outer membrane esterase of Salmonella typhimurium.  Ref: 10559163 J Bacteriol, 1999
